# Impact of post fermentation cooling patterns on fatty acid profile, lipid oxidation and antioxidant features of cow and buffalo milk set yoghurt

**DOI:** 10.1186/s12944-020-01263-1

**Published:** 2020-04-15

**Authors:** Imran Taj Khan, Muhammad Nadeem, Muhammad Imran, Anjum Khalique

**Affiliations:** 1 Department of Dairy Technology, University of Veterinary and Animal Sciences, Lahore, Punjab Pakistan; 2 Institute of Home and Food Sciences, Faculty of Life Sciences, Government College University, Faisalabad, Punjab Pakistan; 3 Department of Animal Nutrition, University of Veterinary and Animal Sciences, Lahore, Punjab Pakistan

**Keywords:** Cow and Buffalo yoghurt, Post fermentation cooling patterns, Antioxidant capacity, Lipid oxidation

## Abstract

**Background:**

In the manufacturing of set yoghurt, after reaching 4.6 pH, post fermentation cooling is applied to stop the bacterial activity. Depending upon the required textural and flavor attributes, one phase and two phase cooling patterns are accordingly selected. In one phase cooling, temperature of the yoghurt is rapidly decreased below 10 °C using blast freezing and then it is gradually dropped to 4-5 °C. In two phase cooling, temperature of yogurt is rapidly decreased to less than 20 °C and then it is gradually decreased to 4-5 °C. These cooling phases have a significant impact on textural and flavor perspectives of yoghurt. It is necessary to study the impact of industrially adopted cooling patterns on fatty acid profile, antioxidant characteristics, lipid oxidation and sensory characteristics of cow and buffalo milk set yoghurt.

**Methods:**

This experiment was organized in a completely randomized design and every treatment was replicated five times to minimize the variation. Whole cow and buffalo milk without any standardization were converted to set yoghurt (400 g cups) using *Strepotococcus thermophillus* and *Lactobacillus bulgaricus* as starter bacteria. After reaching 4.6 pH, cow and buffalo yoghurt samples were exposed to three different cooling patterns. In first trial, samples of cow and buffalo yoghurt were cooled from 43 °C to 25 °C in 1 h and finally cooled to 4-5 °C in another hr. (T_1_). In second trial, samples were cooled from 43 °C to 18 °C in 1 hr. and finally cooled down to 4-5 °C in another 1 h. (T_2_). In third trial, samples were cooled from 43 °C to 4-5 °C in 2 h (T_1_). Alteration in fatty acid profile, total antioxidant capacity, reducing power, free fatty acids, peroxide value, conjugated dienes, vitamin A, E, color and flavor of cow and buffalo yoghurt samples were assessed for 20 days at the frequency of 10 days.

**Results:**

All the three cooling patterns had a non-significant effect on compositional attributes of yoghurt. Buffalo milk yogurt had higher percentage of fat, protein and total solids than yoghurt prepared from cow milk (*p* < 0.05). At zero day, DPPH free radical scavenging activity of T_2_ and T_3_ was significantly higher than T_1_. This may be due to the longer exposure of T_1_ at relatively higher temperature than T_2_ and T_3_. Effect of storage period up to 10 days was non-significant in T_2_ and T_3._ Reducing power of cow and buffalo milk yoghurt was also significantly affected by the cooling patterns applied. Reducing power of T_2_ and T_3_ was considerably higher than T_1_ (*p* < 0.05). At zero-day, total antioxidant capacity of cow and buffalo milk yoghurt in T_3_ was 42.6 and 61.4%, respectively. At zero day, total antioxidant capacity of T_2_ and T_3_ was significantly higher than T_1_. Effect of storage on total antioxidant capacity of T_2_ and T_3_ remained non-significant till 10 days of storage. At zero day, the impact of cooling patterns on fatty acid profile of T_1_, T_2_ and T_3_ was non-significant, whereas, storage period had a marked impact on fatty acid profile. After 10 days, T_1_ was considerably different in fatty acids from T_2_ and T_3_. After 10 days of storage of cow milk yoghurt in T_1_, concentration of C_4:0_, C_6:0_, C_8:0_, C_10:0_, C_12:0_, C_14:0_, C_16:0_, C_18:0_, C_18:1_ and C_18:2_ decreased by 0.1, 0.11, 0.09, 0.07, 0.21, 0.38, 0.28, 0.27, 0.44 and 0.06%, respectively. Cow milk yoghurt in T_1_ after 10 days of storage, concentration of C_4:0_, C_6:0_, C_8:0_, C_10:0_, C_12:0_, C_14:0_, C_16:0_, C_18:0_, C_18:1_ and C_18:2_ decreased by 0.07, 0.15, 0.04, 0.17, 0.20, 0.34, 0.27, 0.36 and 0.04%, respectively. After 10 days of storage in T_2_ and T_3_, loss of fatty acids was 1.2 and 3.61% from C_4:0_ to C_10:0_, respectively. Milk type had no effect on peroxide value of yoghurt. Cooling of cow and buffalo yoghurt from 43 °C to 25 °C had a pronounced effect on peroxide value. At zero day, peroxide values of cow and buffalo yoghurt in T_1_ were 0.32 and 0.33 (MeqO_2_/kg). At zero day, peroxide value of cow and buffalo yoghurt in T_2_ were 0.24 and 0.26 (MeqO_2_/kg). At zero day, peroxide value cow and buffalo yoghurt in T_3_ were 0.23 and 0.25 (MeqO_2_/kg). Cooling patterns i.e. from 43 °C to 25, 18 and 5 °C (T_1_, T_2_ and T_3_) had a significant effect on the amount of vitamin A and E. Concentration of vitamin A and E in T_1_ were significantly less than T_2_ and T_3_. Cooling patterns had a significant effect on texture, T_1_ had a thick texture with higher viscosity as compared to T_2_ and T_3_. Thickness of yoghurt was in the order of T_1_ > T_2_ > T_3_ with no difference in color and flavor score till 10 days of storage.

**Conclusion:**

Results of current investigation indicated that milk type and post fermentation cooling patterns had a pronounced effect on antioxidant characteristics, fatty acid profile, lipid oxidation and textural characteristics of yoghurt. Buffalo milk based yoghurt had more fat, protein, higher antioxidant capacity and vitamin content. Antioxidant and sensory characteristics of T_1_ were optimum till 10 days of storage.

## Background

Milk and fermented dairy products are regarded as basic foods for human nutrition and these products can also assist the body to counter the oxidants. Fermented dairy products are perceived to have more health benefits as compared to milk. Yoghurt is popular all over the world and its chemical and sensory characteristics are largely determined by the source of milk. All over the world, cow milk is the biggest source of milk, about 12% milk is produced by the buffaloes. Chemical composition of cow and buffalo milk is different from each other and latter has higher concentration of fat, protein, lactose and total solids with more white appearance [1]. Buffalo milk has lower concentration of cholesterol as compared to cow milk [2]. All over the world, most of the fermented dairy products are prepared from cow milk, buffalo milk is also highly appropriate for the manufacturing of value added dairy products [3]. Earlier investigations have shown that milk type has a major effect on physical, chemical and sensory prospects of fermented dairy products [4]. Yoghurt is one the most commonly used fermented dairy products throughout the world and *streptococcus thermophilus* and *lactobacillus delbrueckii* ssp. *Bulgaricus* are used as starter culture [5]. Functional value of fermented dairy products is more than native milk and these have more calcium, potassium, riboflavin, thiamin, vitamin B_12_, folic acid and vitamin B_6_ [6]. Yoghurt also contains bioactive peptides which have anti-carcinogenic and antioxidant properties [7]. Milk and dairy products are the fascinating source of antioxidants and whey protein are the most significant source of antioxidants in milk [8]. Milk contains two different types of antioxidants mechanisms i.e. water soluble antioxidants and fat soluble antioxidants. Former is comprised of vitamin C, whey proteins, zinc, selenium etc. and the latter is constituted by tocopherols, retinol, carotenoids, thiols etc. [9]. For example, in the human nutrition, milk and dairy products deliver casein, whey, minerals, vitamins and carotenoids and all these compounds are endowed with antioxidant activity [10]. Antioxidant activity of milk and dairy products may also be due to the existence of plant derived phenols which gain their entry into milk by the feed [11]. Khan et al. [12] also found phenolic compounds and flavonoids in cow and buffalo milk. Several bio-active peptides have been isolated from fermented dairy products and most of them are produced during the process of fermentation and these have strong antioxidant activity [13]. Some enzymes of milk origin, such as glutathione peroxidase and catalase, have antioxidant activity [14]. For example, conversion of hydrogen peroxide to water can be inhibited the antioxidant activity of tripeptide glutathione. Carnosine (β-alanyl-L-histidine, a dipeptide is present skeletal muscles and brain and it may perform antioxidant activities to inhibit free radicals and pro-oxidant activities of metals [15]. Antioxidant systems of milk can help to keep the activities of free radicals under control and these can also aid to inhibit the auto-oxidation [16]. Human body constantly produces free radicals and reactive oxygen species and foods also harbor several types of pro-oxidants which may cause damage to body and food systems. However, these may be inhibited by the antioxidants [17]. Foods should contain enough concentration of antioxidants to safeguard the body from oxidative stresses. Traditional functional foods such as yoghurt contain antioxidant properties; however, the antioxidant properties are significantly affected by the processing conditions such as long pasteurization, homogenization, incubation temperature, starter cultures and post fermentation cooling practices etc. To stop the fermentation process, yoghurt is cooled down, dairy industries adopt different cooling regimes to have desired textural and flavor characteristics and these may be referred as cooling phases. In one phase cooling, temperature of yoghurt is rapidly decreased to less than 10 °C followed by slow cooling to 5 °C. In two phase cooling, temperature of yoghurt is rapidly decreased to less than 18 °C, followed by slow cooling till 5 °C. Both cooling regimes have pronounced effect on flavor and textural characteristics of yoghurt. In two phase cooling, exposure of yoghurt at relatively higher temperature may induce lipid oxidation. Auto-oxidation can lead to the development of sharp unpleasant flavor and deterioration of nutritional profile. Dairy industries use different patterns for the cooling of yoghurt. Impact of post fermentation cooling patterns on antioxidant capacity and lipid oxidation in cow and buffalo yoghurt is not previously investigated. This study may also be helpful in the further standardization of yoghurt technology for the better operation of dairy industries. This study intended to investigate that how the fatty acids and antioxidant characteristics of bovine milk yoghurt are influenced by the post fermentation cooling patterns by the use of advanced and traditional analytical techniques.

## Methods

### Materials

Cow and buffalo milk was obtained from a farm. For analytical work, HPLC grade chemicals were used and procured from Sigma Chemical Co. (St. Louis, MO, USA). Yoghurt starter culture was purchased from CHR-Hansen, Denmark.

### Manufacturing of yoghurt and experimental plan

This experiment was organized in a completely randomized design and every treatment was replicated five times to minimize the variation. Whole cow and buffalo milk without any standardization were converted to set yoghurt (400 g cups) using *Strepotococcus thermophillus* and *Lactobacillus bulgaricus* as starter bacteria. After reaching 4.6 pH, cow and buffalo yoghurt samples were exposed to three different cooling patterns. In first trial, samples of cow and buffalo yoghurt were cooled from 43 °C to 25 °C in 1 h and finally cooled to 4-5 °C in another hr. (T1). In second trial, samples were cooled from 43 °C to 18 °C in 1 h. and finally cooled down to 4-5 °C in another 1 h. (T_2_). In third trial, samples were cooled from 43 °C to 4-5 °C in 2 h (T_3_). Alteration in fatty acid profile, total antioxidant capacity, reducing power, free fatty acids, peroxide value, conjugated dienes, vitamin A, E, color and flavor of cow and buffalo yoghurt samples were assessed for 20 days at the frequency of 10 days.

### Composition of cow, buffalo milk and yoghurt

Chemical composition of cow and buffalo milk was determined on a lactoscan. Fat, protein and total solids were determined for 20 days at the frequency of 10 days using the standard protocols [18].

### Antioxidant characterization of yoghurt

#### Total antioxidant capacity

For the estimation of total antioxidant capacity, spectrophotometric assay was used. Absorbance of the sample, blank and series of standards were recorded at 695 nm and it was expressed in ascorbic acid standard Equi/g [19].

#### DPPH radical scavenging activity

For the determination of DPPH radical scavenging activities in the cow and buffalo yogurt samples, DPPH solution of 192 μL of 50 μM was added to the sample (48 μL). After the mixture, the mixed solution was wrapped with aluminum foil and kept at room temperature in the dark 30 min. 48 μL distilled water was added to 192 μL of 50 μM DPPH in the control sample for standard ascorbic acid. Absorbance of the samples for determination of DPPH was measured at 515 nm on spectrophotometer [20].

#### Reducing power

The method described by Santos et al. [21] was slightly modified for the determination of total reducing power of the yogurt samples. Different concentration of the yogurt samples (0.25 mL) were taken and it was mixed with 0.2 M phosphate buffer (pH 6.7) and 0.25 mL of K_3_Fe (CN)_6_, 1%. The obtained mixture was incubated for 20 min in a water bath at a temperature of 50 °C and 0.25 mL of 10% (w/v) trichloroacetic acid solution was added to stop the reaction. The mixed solution was centrifuged for 10 min at 3000 rpm. Supernatant was obtained, 0.5 ml each supernatant, FeCl_3_ (0.1 ml) and distilled water were mixed and kept for 10 min. The absorbance for determination of ferric reducing power was assessed at 700 nm on spectrophotometer. Gallic acid was used as standard curve of μg GAE/100 ml.

#### Lipid oxidation

For determination of lipid oxidation in set yoghurt samples cooled by two different patterns, free fatty acid, peroxide value and anisidine value were determined at storage days of 0, 10 and 20 days using the standard method of AOCS [22].

#### Vitamin A

The determination of vitamin A in yoghurt samples was conducted by taking 20 g of sample and mixed it with 5 ml of ammonia (25%) and further 20 ml ethanol (96%). BHT at the rate of 0.0025% was added to the extracted supernatant and after that the solution was converted to rotary evaporator for further solvent evaporation at temperature of 35 °C. 30 ml potassium hydroxide in 5% ethanol was used to further saponify the supernatant which was followed by further extraction by the help of *n*-hexane. Sample was concentrated with rotary evaporator and injection volume was 20 ml (HPLC, Shimadzu) [23].

#### Vitamin E

For determination of vitamin E in yogurt samples, first fat was extracted from the samples via standard method [24]. HPLC was used for the estimation of vitamin E concentration in yoghurt samples [25].

#### Fatty acid profile of yoghurt

Fatty acids of yoghurt samples analysis were performed according to the method described by Khas-Erdene et al. [26] and were analyzed using a GC-MS 8790 B gas chromatograph (Agilent, Technologies) fitted with a flame-ionization detector. These samples containing methyl esters in hexane (1 μL) were injected through the split injection port (50,1) onto an HP-88 fused silica 100 mm × 0.25 mm column, 0.20 μm film (Agilent, Agilent Technologies). The oven temperature was initially set at 120 °C for 10 min and was then increased to 230 °C at a rate of 10 °C per min and held at that temperature for 35 min. The injector and detector temperatures were maintained at 250 and 300 °C, respectively and the total run time was 44 min. FAME 37 (Sigmal Aldrich) were used as internal standards. Fatty acids were reported as mg per hundred grams of FAME [27].

#### Sensory evaluation

Sensory evaluation of yoghurt samples was performed by a panel of ten trained judges in a well-ventilated and illuminated sensory evaluation laboratory. Yoghurt samples were evaluated for color, flavor and texture on a 9 point scale, treatments and storage intervals having higher color, flavor and texture were regarded perfect [28].

### Statistical analysis

Experiment was arranged in a completely Randomized Design. For more accuracy in data, every treatment was repeated at least five times with triplicate analysis of all samples. For the determination of effect of treatments storage and their interaction, data were analyzed by two way analysis of variance and LSD test was used to determine the significant difference among the means using SAS 9.1 software [29].

## Results

The results regarding chemical composition of cow and buffalo milk yoghurt cooled by one phase and two phase cooling patterns are shown in Table 1. Fat, protein, lactose, ash, total solids in cow milk were 4.1, 3.2, 4.65, 0.74%, respectively. Fat, protein, lactose, ash, total solids in buffalo milk were 6.2, 3.4, 4.81 and 0.82%, respectively. Cooling of yoghurt in one phase and two phase cooling had no effect on compositional attributes of yoghurt. Milk type had a pronounced effect on compositional attributes of yoghurt as fat, protein and total solids of buffalo milk yoghurt were higher than yoghurt prepared from cow milk (*p* < 0.05). Effect of storage duration (4 °C) on compositional attributes of yoghurt was also assessed for 20 days. All the analysis intervals indicated that storage temperature had non-significant effect on both types of yoghurt. In current investigation, DPPH assay, reducing power and total antioxidant capacity were used to determine the effect of one phase and two phase cooling patterns on antioxidant status of yoghurt. Value of DPPH assay for cow and buffalo yoghurt was 28.4 and 34.5%, respectively. At zero day, DPPH free radical scavenging activity of T_2_ and T_3_ was significantly higher than T_1_. Effect of storage period up to 10 days was non-significant in T_2_ and T_3._ After 10 days of storage while DPPH free radical scavenging activity of T_1_ was significantly less than the initial values recorded at zero days. After 20 days of storage, DPPH free radical scavenging activity of T_2_ and T_3_ were more than T_1_ and significantly less than the values recorded at 0 and 10 days of storage. Table 2 describes the impact of three different cooling patterns on reducing power of cow and buffalo milk yoghurt. Freshly prepared buffalo yoghurt in all the three treatments had higher reducing power than cow milk yoghurt. At zero day, reducing power of cow and buffalo milk yoghurt was also significantly affected by the cooling patterns applied. Reducing power of T_2_ and T_3_ was considerably higher than T_1_ (*p* < 0.05). Reducing power of T_2_ and T_3_ remain unchanged till 10 days of storage period. In T_1_, estimation of reducing power at 10 and 20 days of storage indicated a significant decline. Table 2 describes the effect of milk type, three different cooling patterns and storage duration on total antioxidant capacity of yoghurt. At zero-day, total antioxidant capacity of cow and buffalo milk yoghurt was 42.6 and 61.4%, respectively. At zero day, total antioxidant capacity of T_2_ and T_3_ was significantly higher than T_1_. Effect of storage on total antioxidant capacity of T_2_ and T_3_ remained non-significant till 10 days of storage. Determination of total antioxidant capacity in T_1_ at 10 and 20 days of storage in both cow and buffalo milk yoghurt showed a significant decline.

**Table 1:** Effect of post fermentation cooling patterns on composition of yoghurt

Cooling Patterns	Days		Cow Milk Yoghurt				Buffalo Milk Yoghurt				
		Fat%	Protein%	TS%	Lactose%	Ash%	Fat%	Protein%	TS%	Lactose%	Ash%
T_1_	0	4.11 ± 0.04^b^	3.21 ± 0.02^a^	8.52 ± 0.05^b^	4.62 ± 0.04^a^	0.72 ± 0.01^a^	6.27 ± 0.03^a^	3.42 ± 0.07^a^	15.68 ± 0.09^a^	4.85 ± 0.04^a^	0.84 ± 0.01^a^
	10	4.11 ± 0.07^b^	3.20 ± 0.07^a^	8.50 ± 0.11^b^	4.60 ± 0.02^a^	0.71 ± 0.02^a^	6.26 ± 0.09^a^	3.39 ± 0.01^a^	15.65 ± 0.17^a^	4.82 ± 0.02^a^	0.83 ± 0.02^a^
	20	4.08 ± 0.05^b^	3.18 ± 0.05^a^	8.48 ± 0.06^b^	4.54 ± 0.01^b^	0.70 ± 0.01^a^	6.22 ± 0.03^a^	3.34 ± 0.02^a^	15.62 ± 0.21^a^	4.74 ± 0.05^b^	0.82 ± 0.01^a^
T_2_	0	4.11 ± 0.04^b^	3.21 ± 0.02^a^	8.52 ± 0.05^b^	4.65 ± 0.02^a^	0.73 ± 0.01a	6.27 ± 0.03^a^	3.42 ± 0.07^a^	15.68 ± 0.09^a^	4.83 ± 0.01^a^	0.83 ± 0.01^a^
	10	4.09 ± 0.03^b^	3.18 ± 0.01^a^	8.51 ± 0.12^b^	4.63 ± 0.01^a^	0.72 ± 0.02a	6.25 ± 0.09^a^	3.41 ± 0.05^a^	15.66 ± 0.16^a^	4.84 ± 0.06^a^	0.82 ± 0.02^a^
	20	4.04 ± 0.05^b^	3.17 ± 0.06^a^	8.47 ± 0.14^b^	4.52 ± 0.04^b^	0.71 ± 0.01a	6.22 ± 0.13^a^	3.40 ± 0.02^a^	15.57 ± 0.18^a^	4.75 ± 0.02^b^	0.80 ± 0.02^a^
T_3_	0	4.11 ± 0.04^b^	3.21 ± 0.02^a^	8.52 ± 0.05^b^	4.66 ± 0.02^a^	0.72 ± 0.01^a^	6.27 ± 0.03^a^	3.42 ± 0.07^a^	15.68 ± 0.09^a^	4.86 ± 0.03^a^	0.82 ± 0.03^a^
	10	4.09 ± 0.10^b^	3.18 ± 0.04^a^	8.50 ± 0.03^b^	4.62 ± 0.03^a^	0.74 ± 0.02^a^	6.25 ± 0.11^a^	3.39 ± 0.02^a^	15.65 ± 0.12^a^	4.81 ± 0.02^a^	0.82 ± 0.01^a^
	20	4.07 ± 0.13^b^	3.17 ± 0.09^a^	8.48 ± 0.06^b^	4.51 ± 0.01^b^	0.73 ± 0.02^a^	6.21 ± 0.13^a^	3.37 ± 0.05^a^	15.61 ± 0.07^a^	4.72 ± 0.01^a^	0.81 ± 0.02^a^

*TS* Total Solids

Within the column of a parameter, means expressed by same letter are statistically non-significant (*p* > 0.05)

**Detail of Treatments**

T_1_: Cow and buffalo yoghurt cooled from 43 °C to 25 °C in 1 h and finally cooled to 4-5 °C in another 1 h

T_2_: Cow and buffalo yoghurt cooled from 43 °C to 18 °C in 1 h and finally cooled to 4-5 °C in another 1 h

T_3_: Cow and buffalo yoghurt were cooled from 43 °C to 5 °C in 2 h

**Table 2:** Effect of storage temperature on antioxidant capacity of yoghurt

Cooling Pattern	Days	Cow Milk Yoghurt			Buffalo Milk Yoghurt		
		DPPH%	RP	TAC%	DPPH%	RP	TAC%
T_1_	0	26.4 ± 0.22^c^	8.11 ± 0.34^d^	39.8 ± 0.83^d^	32.3 ± 0.63^a^	13.1 ± 0.39^b^	59.4 ± 0.94^a^
	10	25.3 ± 0.15^c^	7.61 ± 0.13^d^	38.9 ± 0.57^d^	31.6 ± 0.48^b^	11.2 ± 0.73^b^	57.3 ± 1.29^b^
	20	21.5 ± 0.36^e^	3.22 ± 0.16^e^	28.1 ± 0.33^f^	24.2 ± 0.61^c^	8.51 ± 0.24^c^	48.6 ± 0.44^d^
T_2_	0	28.1 ± 0.22^c^	8.65 ± 0.34^c^	41.9 ± 0.83^d^	34.2 ± 0.63^a^	13.8 ± 0.39^a^	60.9 ± 0.94^a^
	10	28.1 ± 0.17^c^	8.63 ± 0.14^c^	42.1 ± 0.77^d^	33.7 ± 0.48^a^	13.9 ± 0.16^a^	60.1 ± 0.72^a^
	20	22.2 ± 0.15^d^	7.51 ± 0.12^d^	38.8 ± 0.68^e^	31.8 ± 0.55^b^	11.8 ± 0.21^b^	56.3 ± 1.12^c^
T_3_	0	28.4 ± 0.22^c^	8.71 ± 0.34^c^	42.6 ± 0.83^d^	34.5 ± 0.63^a^	14.2 ± 0.39^a^	61.4 ± 0.94^a^
	10	28.2 ± 0.19^c^	8.65 ± 0.04^c^	42.4 ± 0.54^d^	34.2 ± 0.47^a^	14.1 ± 0.28^a^	60.9 ± 1.19^a^
	20	26.1 ± 0.33^d^	7.56 ± 0.08^d^	40.1 ± 0.38^e^	32.8 ± 0.26^b^	12.8 ± 0.45^b^	58.5 ± 0.84^b^

Within the two columns of a parameter, means expressed by same letter are statistically non-significant (*p* > 0.05)

*DPPH* 2,2-diphenyl-1-picrylhydrazyl, *RP* Reducing Power, *TAC* Total Antioxidant Capacity

**Detail of Treatments**

T_1_: Cow and buffalo yoghurt cooled from 43 °C to 25 °C in 1 h and finally cooled to 4-5 °C in another 1 h

T_2_: Cow and buffalo yoghurt cooled from 43 °C to 18 °C in 1 h and finally cooled to 4-5 °C in another 1 h

T_3_: Cow and buffalo yoghurt were cooled from 43 °C to 5 °C in 2 h

In this investigation, impact of cooling patterns was investigated on fatty acid profile of cow and buffalo milk based set yoghurt. Yoghurt samples were fermented at 43 °C and cooled down to 25 °C, 18 °C and 4 °C in two hours. Fatty acid profile of cow and buffalo yoghurt samples cooled down from 43 °C to 25 °C was different from the other cooling patterns (T_1_). The difference in fatty acid profile of cow and buffalo milk was also evident, when converted to yoghurt. At zero day, the effect of cooling patterns on fatty acid profile of T_1_, T_2_ and T_3_ was non-significant, whereas, storage period had a significant effect on fatty acid profile (Table 3). After 10 days, fatty acid profile of T_1_ was considerably different from T_2_ and T_3_. After 10 days of storage of cow milk yoghurt in T_1_, concentration of C_4:0_, C_6:0_, C_8:0_, C_10:0_, C_12:0_, C_14:0_, C_16:0_, C_18:0_, C_18:1_ and C_18:2_ decreased by 0.1, 0.11, 0.09, 0.07, 0.21, 0.38, 0.28, 0.27, 0.44 and 0.06%, respectively. After 10 days of storage of cow milk yoghurt in T_1_, concentration of C_4:0_, C_6:0_, C_8:0_, C_10:0_, C_12:0_, C_14:0_, C_16:0_, C_18:0_, C_18:1_ and C_18:2_ decreased by 0.07, 0.15, 0.04, 0.17, 0.20, 0.34, 0.27, 0.36 and 0.04%, respectively. After 10 days of storage in T_2_ and T_3_, loss of fatty acids from C_4:0_ to C_10:0_ was 1.2 and 3.61%, respectively. After 20 days of storage in T_1_, T_2_ and T_3_, the loss of short-chain fatty acids in cow milk yoghurt was 1.48, 12.32 and 19.46%, respectively. After 10 days of storage in T_1_, T_2_ and T_3_, the loss of medium-chain fatty acids in cow milk yoghurt was 0.34 and 4.66%, respectively. After 20 days of storage in T_2_ and T_3_, the loss of medium-chain fatty acids in cow milk yoghurt was 4.95 and 10.58%, respectively. After 10 days of storage in T_2_ and T_3_, the loss of unsaturated fatty acids in cow milk yoghurt was 0.97 and 4.33%, respectively. After 20 days of storage in T_2_ and T_3_, the loss of unsaturated fatty acids in cow milk yoghurt was 5.65 and 11.2%, respectively. After 10 days of storage in T_2_ and T_3_, the loss of short-chain fatty acids in buffalo milk yoghurt was 1.88 and 7.1%, respectively. After 10 days of storage in T_2_ and T_3_, the loss of medium-chain fatty acids in buffalo milk yoghurt was 0.6 and 3.26%, respectively. After 10 days of storage in T_2_ and T_3_, the loss of unsaturated fatty acids in buffalo milk yoghurt was 0.53 and 4.28%, respectively. After 20 days of storage in T_2_ and T_3_, the loss of short-chain fatty acids in buffalo milk yoghurt was 8.77 and 14.81%, respectively. After 20 days of storage in T_2_ and T_3_, the loss of medium-chain fatty acids in buffalo milk yoghurt was 4.57 and 9.12%, respectively. After 20 days of storage in T_2_ and T_3_, the loss of unsaturated fatty acids in buffalo milk yoghurt was 4.92 and 25.47%, respectively.

**Table 3:** Effect of Post Fermentation Cooling Patterns on Fatty Acid Profile of Yoghurt

Fatty Acid	Milk Origin	Cooling Patterns								
		Cooled from 43 °C to 25 °C in 1 h and further cooled to 4-5 °C in 1 h (T_1_)			Cooled from 43 °C to 18 °C in 1 h and further cooled to 4-5 °C in 1 h (T_2_)			Cooled from 43 °C to 4-5 °C in 2 h (T_3_)		
		0-Day	10 Days	20 Days	0-Day	10 Days	20 Days	0-Day	10 Days	20 Days
C_4:0_	Cow	3.42 ± 0.12^a^	3.32 ± 0.09^b^	3.11 ± 0.01^c^	3.61 ± 0.12^a^	3.60 ± 0.11^a^	3.28 ± 0.15^b^	3.62 ± 0.12^a^	3.61 ± 0.03^a^	3.59 ± 0.08^a^
	Buffalo	4.05 ± 0.05^a^	3.95 ± 0.07^b^	3.61 ± 0.14^c^	4.16 ± 0.05^a^	4.14 ± 0.15^a^	3.92 ± 0.04^b^	4.17 ± 0.05^a^	4.15 ± 0.09^a^	4.11 ± 0.04^a^
C_6:0_	Cow	2.25 ± 0.02^a^	2.14 ± 0.04^b^	1.71 ± 0.08^c^	2.34 ± 0.02^a^	2.32 ± 0.03^a^	1.84 ± 0.02^b^	2.35 ± 0.02^a^	2.34 ± 0.02^a^	2.31 ± 0.01^a^
	Buffalo	4.10 ± 0.05^a^	3.95 ± 0.08^b^	3.38 ± 0.03^c^	4.10 ± 0.05^a^	4.07 ± 0.16^a^	3.62 ± 0.13^b^	4.11 ± 0.05^a^	4.09 ± 0.08^a^	4.06 ± 0.10^a^
C_8:0_	Cow	1.51 ± 0.01^a^	1.42 ± 0.03^b^	1.21 ± 0.10^c^	1.60 ± 0.01^a^	1.58 ± 0.04^a^	1.37 ± 0 .05^b^	1.61 ± 0.01^a^	1.60 ± 0.10^a^	1.58 ± 0.03^a^
	Buffalo	2.63 ± 0.06^a^	2.59 ± 0.11^b^	2.26 ± 0.05^c^	2.77 ± 0.06^a^	2.75 ± 0.02^a^	2.43 ± 0.01^b^	2.78 ± 0.06^a^	2.76 ± 0.02^a^	2.73 ± 0.09^a^
C_10:0_	Cow	3.11 ± 0.07^a^	3.04 ± 0.14^b^	2.66 ± 0.02^c^	3.18 ± 0.07^a^	3.16 ± 0.09^a^	2.97 ± 0.06^b^	3.21 ± 0.07^a^	3.17 ± 0.11^a^	3.15 ± 0.16^a^
	Buffalo	4.15 ± 0.03^a^	4.11 ± 0.12^b^	3.86 ± 0.21^c^	4.32 ± 0.03^a^	4.29 ± 0.15^a^	4.07 ± 0.18^b^	4.33 ± 0.03^a^	4.30 ± 0.13^a^	4.28 ± 0.21^a^
C_12:0_	Cow	3.98 ± 0.06^a^	3.77 ± 0.18^b^	3.59 ± 0.17^c^	4.12 ± 0.06^a^	4.11 ± 0.12^a^	3.88 ± 0.02^b^	4.14 ± 0.06^a^	4.13 ± 0.15^a^	4.11 ± 0.23^a^
	Buffalo	5.05 ± 0.09^a^	4.88 ± 0.20^b^	4.28 ± 0.15^c^	5.20 ± 0.09^a^	5.18 ± 0.13^a^	4.95 ± 0.12^b^	5.22 ± 0.09^a^	5.19 ± 0.16^a^	5.15 ± 0.31^a^
C_14:0_	Cow	11.19 ± 0.12^a^	10.79 ± 0.16^b^	9.71 ± 0.29^c^	11.41 ± 0.12^a^	11.35 ± 0.22^a^	10.61 ± 0.23^b^	11.42 ± 0.12^a^	11.38 ± 0.19^a^	11.27 ± 0.36^a^
	Buffalo	9.66 ± 0.14^a^	9.46 ± 0.26^b^	8.54 ± 0.35^c^	10.33 ± 0.14^a^	10.22 ± 0.18^a^	9.76 ± b0.19^b^	10.34 ± 0.14^a^	10.26 ± 0.18^a^	10.19 ± 0.41^a^
C_16:0_	Cow	27.53 ± 0.33^a^	27.25 ± 0.81^b^	26.29 ± 0.63^c^	28.69 ± 0.33^a^	28.66 ± 0.56^a^	27.59 ± 0.38^b^	28.71 ± 0.33^a^	28.55 ± 0.49^a^	28.42 ± 0.59^a^
	Buffalo	26.29 ± 0.41^a^	25.63 ± 0.16^b^	24.64 ± 0.54^c^	27.26 ± 0.41^a^	27.19 ± 0.40^a^	26.18 ± 0.57^b^	27.29 ± 0.41^a^	27.22 ± 0.55^a^	27.17 ± 0.72^a^
C_18:0_	Cow	8.15 ± 0.16^a^	9.03 ± 0.23^b^	7.53 ± 0.48^c^	9.19 ± 0.16^a^	9.16 ± 0.37^a^	8.54 ± 0.17^b^	9.22 ± 0.16^a^	9.18 ± 0.47^a^	9.16 ± 0.13^a^
	Buffalo	10.17 ± 0.19^a^	10.44 ± 0.38^b^	9.42 ± 0.68^c^	11.34 ± 0.19^a^	11.30 ± 0.14^a^	10.22 ± 0.31^b^	11.36 ± 0.19^a^	11.32 ± 0.67^a^	11.27 ± 0.27^a^
C_18:1_	Cow	21.77 ± 0.15^a^	21.33 ± 0.14^b^	20.16 ± 0.69^c^	22.51 ± 0.15^a^	22.34 ± 0.28^a^	21.64 ± 0.19^b^	22.53 ± 0.15^a^	25.39 ± 0.83^a^	25.26 ± 0.46^a^
	Buffalo	26.48 ± 0.46^a^	26.12 ± 0.56^b^	25.34 ± 0.75^c^	27.44 ± 0.46^a^	27.39 ± 0.54^a^	26.31 ± 0.28^b^	27.48 ± 0.46^a^	27.41 ± 0.35^a^	27.34 ± 0.38^a^
C_18:2_	Cow	0.31 ± 0.01^a^	0.25 ± 0.05^b^	0.15 ± 0.01^c^	0.32 ± 0.01^a^	0.31 ± 0.02^a^	0.24 ± 0.03^b^	0.34 ± 0.01^a^	0.32 ± 0.02^a^	0.31 ± 0.02^a^
	Buffalo	0.45 ± 0.02^a^	0.41 ± 0.08^b^	0.13 ± 0.02^c^	0.53 ± 0.02^a^	0.49 ± 0.01^a^	0.34 ± 0.07^b^	0.55 ± 0.02^a^	0.51 ± 0.01^a^	0.48 ± 0.04^a^

Within the row of a same fatty acid, means having a dissimilar letter are statistically significant (*p* < 0.05)

Cooling of cow and buffalo yoghurt from 43 °C to 25 °C (T_1_) had a significant effect on the magnitude of free fatty acids, while the effect of milk source was non-significant (Table 4). Free fatty acids of cow and buffalo milk yoghurt increased in the storage, in T_1_, T_2_ and T_3_, the effect of storage up to 10 days was non-significant. After 20 days of storage of cow and buffalo yoghurt in T_1_, free fatty acids content were significantly higher than T_2_ and T_3_. After 20 days of in T_1_, free fatty acids of cow and buffalo milk yoghurt were 0.16 and 0.17%, respectively. Milk type had no effect on peroxide value of yoghurt. Cooling of cow and buffalo yoghurt from 43 °C to 25 °C had a pronounced effect on peroxide value. At zero day, peroxide values of cow and buffalo yoghurt in T_1_ were 0.32 and 0.33 (MeqO_2_/kg). At zero day, peroxide value of cow and buffalo yoghurt in T_2_ were 0.24 and 0.26 (MeqO_2_/kg). At zero day, peroxide value of cow and buffalo yoghurt in T_3_ were 0.23 and 0.25 (MeqO_2_/kg). In this investigation, strong correlations were established between the peroxide value and flavor score. Yoghurt samples that have more peroxide value obtained lower flavor score (R^2^ = 0.9885). After 20 days of storage, value of conjugated diens in cow and buffalo milk yoghurt was 0.44 and 0.32, respectively. Conjugated dienes in in T_2_ were 0.11 and 0.12 and in their values in T_3_ were 0.06 and 0.07, respectively. Cooling patterns i.e. from 43 °C to 25, 18 and 5 °C (T_1_, T_2_ and T_3_) had a significant effect on the amount of vitamin A and E. Concentration of vitamin A and E in T_1_ were significantly less than T_2_ and T_3_ (Table 5). Buffalo yoghurt had higher amount of vitamin of A and E as compared to cow yoghurt. In T_1_, the effect of storage period on cow and buffalo yoghurt was significant and estimation of vitamin A and E after 10 days indicated a significant decline in their concentration and even this trend was also evident after 20 days of storage. The effect of different post fermentation cooling patterns on sensory characteristics of cow and buffalo milk yoghurt is explained in Table 6. Freshly prepared buffalo milk had more color score than cow milk yoghurt (*p* < 0.05). The flavor of both cow and buffalo yoghurt was not affected by the type of milk. Cooling patterns i.e. from 43 °C to 25, 18 and 5 °C (T_1_, T_2_ and T_3_) had a significant effect on texture, T_1_ had a thick texture with higher viscosity as compared to T_2_ and T_3_. Thickness of yoghurt was in the order of T_1_ > T_2_ > T_3_. Color and flavor score of cow and buffalo yoghurt cooled by three different cooling patterns decreased after 20 days.

**Table 4:** Lipid oxidation of cow and buffalo milk yoghurt under different cooling patterns

Storage Temperature	Days	Cow Milk Yoghurt			Buffalo Milk Yoghurt		
		FFA	PV	CD	FFA	PV	CD
T_1_	0	0.11 ± 0.01^b^	0.32 ± 0.02^c^	0.07 ± 0.01^e^	0.11 ± 0.02^b^	0.33 ± 0.01^c^	0.08 ± 0.01^e^
	10	0.13 ± 0.03^b^	0.39 ± 0.05^b^	0.21 ± 0.03^c^	0.13 ± 0.01^b^	0.38 ± 0.03^b^	0.18 ± 0.03^c^
	20	0.16 ± 0.02^a^	0.51 ± 0.07^a^	0.44 ± 0.05^a^	0.17 ± 0.03^a^	0.48 ± 0.06^a^	0.32 ± 0.02^b^
T_2_	0	0.08 ± 0.01^c^	0.24 ± 0.02^d^	0.05 ± 0.01^e^	0.10 ± 0.02^c^	0.26 ± 0.01^d^	0.07 ± 0.01^e^
	10	0.09 ± 0.03^c^	0.26 ± 0.05^d^	0.06 ± 0.02^e^	0.12 ± 0.01^b^	0.28 ± 0.04^d^	0.09 ± 0.02^e^
	20	0.10 ± 0.01^b^	0.41 ± 0.03^b^	0.11 ± 0.01^d^	0.14 ± 0.02^b^	0.38 ± 0.05^b^	0.12 ± 0.03^d^
T_3_	0	0.08 ± 0.01^c^	0.23 ± 0.02^d^	0.04 ± 0.01^e^	0.09 ± 0.02^c^	0.25 ± 0.01^d^	0.05 ± 0.01^e^
	10	0.09 ± 0.02^c^	0.25 ± 0.03^d^	0.05 ± 0.02^e^	0.10 ± 0.01^c^	0.27 ± 0.03^d^	0.06 ± 0.02^e^
	20	0.09 ± 0.01^c^	0.26 ± 0.04^d^	0.06 ± 0.01^e^	0.11 ± 0.02^c^	0.28 ± 0.02^d^	0.07 ± 0.01^e^

Within the column of a same parameter, means having a dissimilar letter are statistically significant (*p* < 0.05)

FFA: Free Fatty Acids% (Oleic Acid)

PV: Peroxide Value (MeqO_2_/kg)

CD: Conjugated Dienes

**Detail of Treatments**

T_1_: Cow and buffalo yoghurt cooled from 43 °C to 25 °C in 1 h and finally cooled to 4-5 °C in another 1 h

T_2_: Cow and buffalo yoghurt cooled from 43 °C to 18 °C in 1 h and finally cooled to 4-5 °C in another 1 h

T_3_: Cow and buffalo yoghurt were cooled from 43 °C to 5 °C in 2 h

**Table 5:** Lipid vitamin A and E of cow and buffalo milk yoghurt under different cooling patterns

Cooling Patterns	Days	Cow Milk Yoghurt		Buffalo Milk Yoghurt	
		Vitamin A μg/100 g	Vitamin E mg/100 g	Vitamin A μg/100 g	Vitamin E mg/100 g
T_1_	0	42.19 ± 0.08^d^	0.16 ± 0.03^a^	118.47 ± 0.07^a^	0.88 ± 0.01^a^
	10	44.28 ± 0.44^e^	0.13 ± 0.02^a^	112.56 ± 0.74^b^	0.77 ± 0.03^a^
	20	36.27 ± 0.66^f^	0.07 ± 0.01^b^	93.66 ± 0.34^c^	0.58 ± 0.02^c^
T_2_	0	50.11 ± 0.08^d^	0.20 ± 0.03^a^	124.47 ± 0.06^a^	1.08 ± 0.01^a^
	10	48.98 ± 0.27^d^	0.20 ± 0.04^a^	124.17 ± 0.23^a^	1.09 ± 0.03^a^
	20	44.78 ± 0.39^e^	0.11 ± 0.02^b^	110.37 ± 0.63^b^	0.88 ± 0.10^b^
T_3_	0	55.17 ± 0.08^d^	0.22 ± 0.03^a^	128.47 ± 0.07^a^	1.12 ± 0.01^a^
	10	50.29 ± 0.13^d^	0.21 ± 0.01^a^	126.53 ± 0.82^a^	1.10 ± 0.08^a^
	20	45.66 ± 0.19^e^	0.13 ± 0.02^b^	115.39 ± 0.22^b^	0.92 ± 0.05^b^

Within the column of a same parameter, means having a dissimilar letter are statistically significant (*p* < 0.05)

**Detail of Treatments**

T_1_: Cow and buffalo yoghurt cooled from 43 °C to 25 °C in 1 h and finally cooled to 4-5 °C in another 1 h

T_2_: Cow and buffalo yoghurt cooled from 43 °C to 18 °C in 1 h and finally cooled to 4-5 °C in another 1 h

T_3_: Cow and buffalo yoghurt were cooled from 43 °C to 5 °C in 2 h

**Table 6:** Effect of cooling patterns on sensory characteristics of cow and buffalo milk yoghurt

Cooling Pattern	Days	Cow Milk Yoghurt			Buffalo Milk Yoghurt		
		Color	Flavor	Texture	Color	Flavor	Texture
T_1_	0	7.8 ± 0.03^b^	8.1 ± 0.12^a^	8.4 ± 0.10^a^	8.2 ± 0.05^a^	8.3 ± 0.17^a^	8.3 ± 0.14^a^
	10	7.7 ± 0.02^b^	8.0 ± 0.08^a^	8.1 ± 0.16^a^	8.1 ± 0.03^a^	8.1 ± 0.11^a^	8.1 ± 0.12^a^
	20	7.6 ± 0.06^b^	8.1 ± 0.02^a^	8.1 ± 0.12^a^	8.0 ± 0.12^a^	8.0 ± 0.06^a^	8.0 ± 0.09^a^
T_2_	0	7.8 ± 0.03^b^	8.1 ± 0.12^a^	8.2 ± 0.02^a^	8.2 ± 0.05^a^	8.3 ± 0.17^a^	8.1 ± 0.17^a^
	10	7.8 ± 0.04^b^	7.9 ± 0.16^a^	8.0 ± 0.19^a^	8.0 ± 0.09^a^	8.2 ± 0.04^a^	8.0 ± 0.15^a^
	20	7.2 ± 0.07^b^	7.6 ± 0.14^a^	7.9 ± 0.10^a^	7.9 ± 0.15^a^	8.1 ± 0.08^a^	8.0 ± 0.01^a^
T_3_	0	7.8 ± 0.03^b^	7.8 ± 0.12^a^	7.5 ± 0.03^b^	8.2 ± 0.05^a^	8.1 ± 0.17^a^	7.6 ± 0.09^b^
	10	7.7 ± 0.05^b^	7.0 ± 0.03^c^	7.4 ± 0.08^b^	8.1 ± 0.13^a^	8.2 ± 0.16^c^	7.5 ± 0.07^b^
	20	7.7 ± 0.02^b^	6.3 ± 0.13^d^	7.1 ± 0.06^b^	8.0 ± 0.16^a^	6.5 ± 0.03^d^	7.4 ± 0.05^b^

Within the column of a same parameter, means having a dissimilar letter are statistically significant (*p* < 0.05)

**Detail of Treatments**

Cow and buffalo yoghurt cooled from 43 °C to 4-5 °C in 2 h (T_1_)

Cow and buffalo yoghurt cooled from 43 °C to 18 °C in 1 h and finally cooled down to 4-5 °C in another 1 h. (T_2_)

Cow and buffalo yoghurt cooled from 43 °C to 25 °C in 1 h and finally cooled to 4-5 °C in another hr. (T_3_)

## Discussion

The difference in compositional attributes of buffalo milk yoghurt over cow milk yoghurt was due to higher fat, protein and total solids content in parent buffalo milk. Abdullah et al. [30] recorded that compositional attributes of yoghurt like fermented dairy product were not affected by the fermentation. Similar observation was also noted by Ammar et al. [31] who found no remarkable change in chemical composition of yoghurt and substrate milk. The recent studies in 2017 conducted by Fagnini and his colleagues assessed the effect of storage temperature on compositional parameters of milk. The results indicated that storage temperature did not affect chemical composition of yoghurt [32]. For the assessment of antioxidant status of complex food substrates, DPPH free radical scavenging activity is widely used. For the assessment of antioxidant capacity of whey butter, margarine and cheddar cheese, DPPH free radical scavenging assay was used [33–35]. Buffalo milk has higher content of biochemical compounds having antioxidant capacity e.g. vitamin A, E, C, mineral, casein, whey, catalase, glutathione peroxidase and superoxide dismutase as compared to cow milk. Buffalo milk had higher antioxidant activity as compared to cow milk [12]. The higher antioxidant activity in current study may be due to the longer exposure of T_1_ at relatively higher temperature than T_2_ and T_3_. Antioxidant capacity of milk and dairy products depends upon processing and storage temperature [36]. Antioxidant characteristics of dairy and food products have been extensively studied. However, the effect of different cooling patterns on antioxidant characteristics of cow and buffalo milk has not been previously reported. Antioxidant capacity of yoghurt decreased during the storage [36, 37]. Variation in DPPH free radical scavenging activity was observed during the long term ripening of cheese [35]. Campos et al. [38] conducted studies on antioxidant capacity of yogurt for the storage period of 28 days and DPPH antiradical activity assay was used to find anti-oxidative activity of samples. The results showed that antioxidant capacity of yogurt decreased in the storage phase. Studies on post-acidification and stability of antioxidant activities of *Acai* yogurt was studied when stored at 4 °C for 28 days. Results showed that at the end of 28 days, antioxidant activity of yoghurt and fermented milks decreased significantly [39].

Reducing power measures the conversion of oxidation form of Fe+ ^3^ in Fe+ ^2^ as an indication of antioxidant activity. Reducing power assay is important for the determination of reducing power of food systems [40]. For the characterization of antioxidant characteristics of cow and buffalo milk, Khan et al. [12] used reducing power as an important indicator of antioxidant activity. Freshly prepared buffalo yoghurt in all the three treatments had higher reducing power than cow milk yoghurt and this may be attributed to the occurrence of antioxidant substances in higher magnitude in buffalo milk. The lower reducing power in T_1_ can be connected to the relatively higher temperature in the downstream cooling (25 °C). Effect of storage on antioxidant characteristics of yoghurt was monitored for the duration of 21 days and antioxidant characteristics of yoghurt decreased in the storage [36]. Reducing power of yogurt prepared from camel milk decreased in storage period of 21 days [41]. A research conducted on antioxidant activities of fermented beverage kambucha and decline in reducing power was recorded during storage of the product for 14 days [42].

Total antioxidant capacity is defined as collective action of all the antioxidants present in food matrix [43]. Milk and dairy products are complex mixture of vitamins, minerals, proteins and enzyme systems and these chemicals constitutes of milk has antioxidant capacity [44]. With the advancement of knowledge in nutrition and disease, nutritionists suggest consuming traditional functional foods. Yoghurt is one of the most commonly used dairy products in the world with several health benefits. Studies have shown that fermented version of milk products have higher antioxidant capacity than parent milk [45]. Antioxidant characterization of bovine milk yoghurt needs to be elaborated to promote the traditional functional foods. Antioxidant capacity of traditional and commercial yoghurt was compared and former had higher antioxidant capacity as compared to the latter [46]. Irma and Worapot [47] investigated the impact of fermentation, storage temperature on antioxidant status of cow, buffalo and goat milk yoghurt. Results revealed that cow, buffalo and goat milk yoghurts had higher antioxidant capacity as compared to native milks under standard storage temperature (4 °C), and the storage results are matching with the studies conducted on the antioxidant capacities of yogurt fortified with stevia which decreased gradually as the product continues to be in storage of 30 days [48].

Milk fat is one of the most complex fat containing more than two hundred different types of fatty acids and large number of triglycerides [49]. Short-chain fatty acids; butyric, capric, caproic and capryllic have been implicated with typical flavor of milk and its derived products [50]. Fatty acid profile of milk and dairy products may change due to the lipid oxidation [33]. Milk fat contains reasonable amount of unsaturated fatty acids and high temperature processing can lead to lipid oxidation. In current investigation, assessment of fatty acid profile was used as a marker of oxidation status of milk fat. Cooling patterns had a significant impact on fatty acid profile. Effect of storage period on fatty acid profile of dairy products has reported in previous investigations. Fatty acid profile of dairy products largely depends upon the storage temperature [34]. Fatty acid profile was strongly connected with peroxide value and determination frequencies showing extensive breakdown of unsaturated fatty acids showed higher peroxide value [51]. Nadeem et al. [33] reported that fatty acid profile of butter oil significantly changed in the storage period. Fatty acid profile of butter manufactured from low melting point fractions changed during the extended storage period. Fatty acid profile of fermented cream changed during the storage phase [52]. Domagala et al. [53] found a non-significant effect on long chain fatty saturated acids in fermented milk stored at 4 °C for 15 days. Changes were observed in the fatty acid profile of goat milk and yogurt during processing as described by [54]. Similar results about the decrease in tendency of myristic and palmitic acids were also observed in yogurt fatty acid profile when yogurt fat was replaced with rose hip seed oil [55].

For the assessment of lipid oxidation in cow and buffalo milk yoghurt exposed to three different cooling patterns, classical techniques of lipid oxidation measurements were used; these included the free fatty acids, peroxide value and conjugated dienes. These techniques of lipid oxidation measurement are widely used and recently reported in literature [34, 35, 51]. Milk and dairy products contain about 23–25% unsaturated fatty acids which are susceptible to auto-oxidation. For the assessment of lipid oxidation and shelf life anticipation, accelerated oxidation technique is commonly used for oils, fats and several other foods. However, these techniques are not usually employed for the anticipation of oxidative behavior of dairy products especially yoghurt. The non-frequent use of accelerated oxidation (Induction Period) for the dairy products may be due to the incompatibility of the dairy products with this technique. Therefore, classical techniques are commonly used for the assessment of oxidation status of milk and dairy products. Free fatty acids are the product of hydrolysis which is catalyzed by lipases (indigenous and bacterial), moisture content and metal ions etc. Free fatty acids may lead to the development of off flavor in dairy products and foods. Free fatty acids should be within the allowable limit of European Union i.e. 0.20% max (oleic acid). Lipase activity in food matrix largely depends upon the processing and storage temperature and higher the processing/ storage temperature, greater would be the lipase activity and vice versa. The results of this investigation are in line with the findings of Nadeem et al. [33] who reported that free fatty acids of margarine did not increase when stored at 4-5 °C. Nadeem et al. [51] also observed that free fatty acids of butter increased during the storage. The results for free fatty acids are in agreement with studies conducted by Diaz et al. [56]. Peroxide value assay is conducted to have information on the oxidation status of foods. Food system having lower peroxide value is usually perceived to have reasonable oxidative stability and shelf life [57]. Nadeem et al. [58] observed that peroxide value of butter increased as the storage period increased and a strong connection between peroxide value and flavor score was also established. Okullo et al. [59] found that peroxide value of shea butter increased during storage. Peroxide value of fermented cream was influenced by the storage period [60]. Conjugated dienes represent the primary oxidation products generated in food systems due to the breakdown of fatty acids. The current data is in line with the conclusion obtained by De Nunzio et al. [61] that concentration of conjugated dienes was affected by the processing conditions.

Estimation of vitamin A and E was done in this study due to their nutritional and antioxidant perspectives in milk and dairy products. In changing life styles, it is extremely important to consume a functional diet that contains bioactive compounds. Presently, most of the research work is focused on the nutritional and antioxidant characterization of traditional functional foods. Antioxidant potential of traditional food is being discovered to guide the food industries to develop functional foods. Most of the foods and dairy products are not immediately consumed rather these are stored from few days to few weeks for sale and/or consumption. Storage temperature of super market and house hold refrigeration facilities considerably varies, therefore, antioxidant characterization of cow and buffalo yoghurt was assessed. The higher antioxidant capacity of buffalo milk over the cow milk is reported [12]. The decline in vitamin A and E in T_1_ was due to the longer exposure of yoghurt at relatively higher temperature. Previous studies conducted by Andino and Daniel [62] revealed that during 4 weeks of refrigerated storage, vitamins (α-tocopherol) concentration in plain yogurt decrease promptly. This research results are in line with studies conducted by Bonczar et al. [63] which shows that vitamin C and E concentration in milk and fermented milk products including yogurt decreased significantly after storage for 14 days.

With respect to sensory parameters, the greater score of buffalo milk was probably due to the rich creamy white color. This study is supported with the findings of Sfakianakis and Tzia [64] who reported that phase cooling had a great effect on viscosity of yoghurt. Similar trends were subjected by Falade et al. [65] that sensory characteristics of fortified yoghurt decreased during the storage phase. The deterioration in flavor score in cow and milk yoghurt at different cooling patterns was due to the auto-oxidation and generation of oxidation products such as aldehydes, ketones and alcohols etc. For the production of thick yoghurt, it may be cooled from 43 °C to 25 °C in 1 h and then to 5 °C in another 1 h., but it should be consumed within the few days.

## Conclusion

The results of this investigation suggested that post fermentation cooling patterns and milk type significantly affected the antioxidant characteristics and vitamin content of yoghurt. T_2_ and T_3_ had higher DPPH free radical scavenging activity, reducing power and total antioxidant capacity than T_1_. After 20 days of storage, peroxide value of T_1_ was significantly higher than T_2_ and T_3_. Texture score of T_1_ was more than T_2_ and T_3_ with no difference in color and flavor score till 10 days of storage period. It can be concluded that post fermentation cooling patterns may be applied according to the consumers requirements. If method of post fermentation cooling (cooling from 43 °C to 25 °C in 1 h. and cooling to 4-5 °C in another 1 h) is applied, thick yoghurt may be prepared but it should be consumed within 10 days of storage period for optimum antioxidant and sensory characteristics.

## Data Availability

The dataset supporting the conclusions of this article is included within the article.

## Abbreviations

DPPH: 2,2-Diphenyl-1-Picrylhydrazyl
HPLC: High Performance Liquid Chromatography
GC: Gas Chromatography
TAC: Total Antioxidant Capacity
RP: Reducing Power
